# RNA-seq analysis identifies key genes and signaling pathways involved in androgen promotion of sebaceous gland proliferation in Hetian sheep

**DOI:** 10.1038/s41598-025-08837-y

**Published:** 2025-07-02

**Authors:** Zhiya Feng, Zhigang Li, Wanwan Peng, Jianping Zhang, Chunyang Li, Ruijun Shi, Shuwei Li

**Affiliations:** 1 State Key Laboratory Incubation Base for Conservation and Utilization of Bio-Resource in Tarim Basin, College of Life Science and Technology, Tarim University, Alar, 843300 China; 2 Collage of Natural Resources and Environment, Northwest A&F University, Yangling, 712100 China

**Keywords:** Hetian sheep, Skin, RNA-seq, Masson staining, GSEA, Computational biology and bioinformatics, Transcriptomics

## Abstract

Sebaceous glands (SGs), essential elements of the skin barrier in sheep, are tightly regulated by the androgen signaling pathway. This study investigated how androgens influence SGs hyperplasia in sheep by combining morphological assessments with transcriptomic analysis. Histological examination revealed a significant increase (*P* < 0.05) in both the number and average size of SGs in the testosterone-treated groups across four concentration gradients compared to the control group. Transcriptome sequencing was performed on fifteen skin samples, revealing that, compared to the control group, 371 genes were upregulated and 115 were downregulated across the four treatment groups. The upregulated genes were predominantly enriched in lipid metabolism pathways, while the downregulated genes were mainly associated with keratin filament structures. Protein-protein interaction (PPI) network analysis identified ten hub genes involved in mitochondrial β-oxidation and lipid synthesis. These findings were further supported by gene set enrichment analysis (GSEA), which indicated significant activation of fatty acid metabolic pathways (*P* < 0.05). The results of this study suggest that androgens may be involved in SGs hyperplasia in sheep by regulating mitochondrial lipid metabolism-related pathways.

**Supplementary Information:**

The online version contains supplementary material available at 10.1038/s41598-025-08837-y.

## Introduction

Hetian sheep, a locally bred, semi-coarse wool type with heterogeneous characteristics, has a long-standing presence in China. Primarily found in Xinjiang, this breed is renowned for its resilience to drought, capacity to use coarse forage efficiently, and high-quality meat production. Moreover, Hetian sheep are highly prized for their exceptional wool fibers, the preferred raw material for crafting traditional Hetian handmade carpets^1^. The skin of Hetian sheep acts as a protective barrier against environmental damage and fluid loss through several key features. Within the skin, hair follicles and sebaceous glands (SGs) together constitute the pilosebaceous unit^2^, which is essential for thermoregulation, protection, and the secretion of sweat and sebum, as well as for sensory and tactile functions^3,4^.

Moreover, the outermost layer of the sebaceous gland contains actively proliferating sebaceous progenitor cells, while the inner layer is composed of sebocytes specialized keratinocytes responsible for sebum production^2^. Mature sebum plays a vital role in maintaining mammalian skin barrier integrity by lubricating hair, minimizing transepidermal water loss, providing water repellency, and aiding in thermoregulation^5^. Furthermore, beyond sebum production, SGs also contribute to innate immunity and androgen metabolism^6^.

SGs are vital components of sheep skin appendages as they are responsible for maintaining the integrity of the skin barrier^7^. Moreover, they also act as target organs for androgen activity^8^. Studies in mammals have shown that androgens like testosterone (T) and dihydrotestosterone (DHT) regulate the development of hair follicles and sebaceous glands through ligand-dependent activation of androgen receptors (ARs)^8^. Androgen exposure promotes the proliferation and differentiation of AR-positive immature sebocytes by upregulating genes specific to sebocyte differentiation. This is accompanied by the downregulation of markers linked to undifferentiated hair follicle cell populations^9^. Thus, androgens are known to stimulate sebum production and affect hair follicle development. However, the specific role of endogenous androgens in sustaining skin function remains incompletely understood, and studies investigating their impact on the skin of livestock are still limited.

A testosterone concentration gradient with five distinct levels was administered to Hetian sheep to assess its effects on skin morphology and structure. To further explore the impact of T on gene expression in the skin, RNA sequencing (RNA-seq) was conducted. The functions of androgen-regulated differentially expressed genes (DEGs) were examined through enrichment analyses to identify key genes and signaling pathways involved in SG growth and development. Eight genes were confirmed by quantitative real-time PCR (qRT-PCR) to validate the transcriptomic data, and two corresponding proteins were detected using tissue immunofluorescence. These findings improve our understanding of androgen action and the molecular mechanisms governing SG function in mammalian skin. Moreover, this study offers valuable insights for identifying potential molecular targets in androgen-dependent skin disorders.

## Materials and methods

### Experimental animals

All experimental procedures were conducted following relevant guidelines and regulations. Fifteen healthy, non-pregnant ewes of similar age (2 ± 0.5 years) and body weight (35 ± 2.5 kg) were sourced from the Hetian Sheep Breeding Farm in Luopu County, Xinjiang. The animals were individually housed in enclosures measuring 2.4 m by 1.2 m, each equipped with feeding troughs and water buckets, at the Laboratory Animal Center of Tarim University. They were maintained under stable natural light cycles, temperature, and feeding conditions, receiving approximately 1.5 kg of dry alfalfa grass and 200 g of corn kernels daily and unrestricted water access. All animal handling and experimental protocols adhered to the ARRIVE guidelines (https://arriveguidelines.org) and were approved by the Ethics Committee of Science and Technology at Tarim University (Approval number: PB20250312001).

### Testosterone (T) treatment and sample collection

Fifteen Hetian sheep were randomly divided into five groups containing three animals. Four groups (TG1–TG4) received T treatments, while the fifth group served as the control (CG). Testosterone (Yuanye Biotechnology Co., Ltd.) was dissolved in corn oil for administration. Over 42 days, sheep in the treatment groups were given intramuscular T injections at doses of 1, 2, 3, or 4 mg/kg body weight every three days, with the injection volume standardized to 4 mL per animal. The control group received equivalent volumes (4 mL) of corn oil on the same schedule. On day 42, back skin samples were collected after shaving and disinfecting the area with iodine. A local anesthetic, procaine hydrochloride (0.5 mL), was injected subcutaneously near the sampling site before obtaining skin biopsies using a 1 cm diameter punch. The collected samples were immediately snap-frozen and stored in liquid nitrogen. Post-biopsy, the wounds were sutured, treated daily with antibiotics, and monitored for one week to ensure proper healing.

### Preparation and morphological analysis of skin tissue sections

Skin samples from the TG and CG groups were fixed in 4% paraformaldehyde for 48 h. Following fixation, the samples underwent dehydration through a graded alcohol series, clearing with xylene and paraffin embedding at 65 °C for 2 h. Tissue embedding was performed using a paraffin embedding machine (EGH50H, Leica, Germany), after which the paraffin blocks were sectioned transversely into 8-µm-thick slices using a microtome (RM2016, Leica, Germany). The sections were then floated on distilled water at 42 °C, mounted onto slides, and baked for 3 h at 55 °C. According to the manufacturer’s protocol, Masson’s staining was carried out on the sections using a commercial kit (G1340, Beijing Solarbio Science & Technology Co., Ltd., China). Images of the stained sections were captured with an optical microscope (Eclipse E200MV, Nikon, Japan) equipped with a microscopic imaging system (DS-Fi3, Nikon, Japan) to quantify the area and number of SGs. Three tissue sections per sheep were examined for morphological analysis, with ten randomly selected 1 mm² microscopic fields containing SGs analyzed in each section. The morphometric module of the imaging software was employed to measure the SG area.

The average SG area was determined by dividing the total measured surface area by the number of SGs. The extent of fibrous collagen in the skin was quantified using ImageJ software (version 1.53; developed by Wayne Rasband, National Institutes of Health, Kensington, MD, USA). All data were normalized and presented as mean ± standard error of the mean (SEM) before statistical analysis was performed with SPSS 23 software (SPSS Inc., IBM, Chicago, IL, USA). One-way ANOVA was followed by multiple comparison tests, and the least significant difference (LSD) analysis was used to assess differences between groups. Statistical significance was defined as *P < 0.05 for moderate differences and **P < 0.01 for highly significant differences.

### RNA extraction, cDNA library construction, and sequencing

Total RNA was extracted from skin samples of both treatment and control groups using TRIzol reagent (Tiangen Biochemical Technology Co., Ltd., Beijing, China) according to the manufacturer’s protocol. RNA quality was initially assessed by electrophoresis on a 1% agarose gel (1× TAE buffer, 150 V, 10 min), followed by purity and concentration measurements using a Nanodrop spectrophotometer (NC2000; Thermo Scientific, Waltham, MA, USA). RNA integrity was further evaluated using a Bioanalyzer 2100 system (Agilent Technologies Inc., Santa Clara, CA, USA) with an RNA Nano 6000 assay kit (5067 − 1511; Agilent Technologies Inc.). For each sample, 3 µg of RNA was used to construct cDNA libraries. Messenger RNA (mRNA) was enriched and purified via magnetic beads with oligo(dT) sequences, then fragmented into approximately 300 bp segments. First-strand cDNA synthesis was performed using random hexamer primers and reverse transcriptase, followed by second-strand synthesis using the first strand as a template. Zhongke New Life (Shanghai, China) conducted library preparation and paired-end sequencing on the Illumina NovaSeq 6000 platform (Illumina Inc., San Diego, CA, USA). The processed sequencing data have been deposited in the NCBI Sequence Read Archive (SRA) under accession number PRJNA1202934.

### Quality control of sequencing data and comparative analyses

Raw sequencing data generated by the Illumina platform were preprocessed using Cutadapt v1.15 and FastQC v0.11.8 with default settings to remove low-quality reads, adapter sequences, poly-N stretches, and reads with an average quality score below Q20. Downstream analyses were conducted on the resulting high-quality clean data. The quality metrics of the transcriptome data are provided in the supplementary materials (Supplementary Table S1). Filtered clean reads were mapped to the Ovis aries reference genome (GCF_002742125.1 Oar_Rambouillet_v1.0) after constructing the reference index with Bowtie v2.2.3. Subsequently, paired-end reads were aligned to the genome using TopHat v2.0.12, and gene-level read counts were quantified using HTSeq v0.6.1.

### Screening of differentially expressed genes (DEGs)

The DEGs were identified using thresholds of |log2(fold change)| > 1 and a q-value < 0.05. Each treatment group was compared to the control group, and volcano plots illustrating the DEGs were generated using OmicStudio tools (https://www.omicstudio.cn/). Common DEGs shared among the four treatment groups were then identified through Venn diagram analysis on the bioinformatics platform (http://www.bioinformatics.com.cn/) and list of common DEGs in the supplementary table (Supplementary Table S2).

### Functional enrichment analysis and protein-protein interaction (PPI) network analysis

Co-expressed DEGs were subjected to expression trend analysis using OmicStudio tools. Afterward, enrichment analysis of these DEGs was performed through the DAVID database (https://david.ncifcrf.gov/) to identify significantly enriched Gene Ontology (GO) terms and Kyoto Encyclopedia of Genes and Genomes (KEGG) pathways^10^. Finally, protein-protein interaction (PPI) network analysis was carried out using the STRING database (https://www.string-db.org/), with network visualizations generated in Cytoscape version 3.8.

### Gene set enrichment analysis (GSEA)

The GSEA was performed on the complete gene sets from each TG and CG group using the GO and KEGG databases. Significant pathway enrichment was defined by a normalized enrichment score (|NES|) > 1, a nominal *P*-value < 0.05, and a false discovery rate (FDR) < 0.25. Pathways that met these criteria were used to generate enrichment score (ES) plots. Moreover, pathways with lower FDR values were selected for further analysis to construct gene-pathway enrichment network maps using OmicStudio tools.

### PCR validation of DEGs by quantitative reverse-transcriptase PCR (qRT-PCR)

To validate the RNA-seq results, eight DEGs were selected for confirmation by qRT-PCR. Primers were designed using the NCBI Primer-BLAST online tool, with details provided in the supplementary file (Supplementary Table S3). Total RNA was extracted from samples of all 15 sheep (*n* = 15) using TRIzol reagent. After quality assessment, RNA concentrations were standardized to 2 µg/µL. cDNA synthesis was carried out using a reverse transcription kit (AE311, TransGen Biotech Co., Ltd., China) following the manufacturer’s instructions, and the resulting cDNA was stored at −20 °C. qPCR was performed on a BYQ6619-651598 instrument (Bioer Technology) using TransStart Tip Green qPCR SuperMix (AQ141, TransGen Biotech Co., Ltd.). Each reaction contained 2 µL of cDNA, 0.5 µL of each primer, 12.5 µL of fluorescence PCR mix, and 9.5 µL of RNase-free water. The cycling conditions included an initial denaturation at 94 °C for 30 s, followed by 40 cycles of denaturation at 94 °C for 5 s and annealing/extension at 60 °C for 30 s. GAPDH served as the internal reference gene for normalization, and relative gene expression levels were calculated using the 2^−ΔΔCt^ method. Statistical analyses were conducted using independent samples t-tests in SPSS 23, with data presented as mean ± SEM.

### Immunofluorescence detection

Skin tissue sections were deparaffinized in xylene and rehydrated through a graded series of alcohols. Antigen retrieval was performed using a citric acid buffer (pH 8.0) and blocking endogenous peroxidase activity with 3% hydrogen peroxide. To prevent nonspecific binding, sections were incubated for 45 min in 5% bovine serum albumin. They were then incubated overnight at 4 °C with rabbit anti-ACSL1 (13989-1-AP, Proteintech Group, Inc., USA) and anti-CERS4 (bs-10189R, Beijing Biosynthesis Biotechnology Co., Ltd., China) antibodies, both diluted 1:200. After washing, the sections were incubated for 60 min at room temperature in the dark with Cy3-conjugated goat anti-rabbit IgG secondary antibody (1:200, AS007, ABclonal Biotechnology Co., Ltd., China). Subsequently, nuclei were stained with DAPI (C0060, Beijing Solarbio Science & Technology Co., Ltd., China) for 10 min, and the slides were mounted using an anti-fade mounting medium (S2110, Beijing Solarbio Science & Technology Co., Ltd.) for observation under an inverted fluorescence microscope (HBO100, Zeiss, Germany). Fluorescence intensity was quantified using ImageJ software.

## Results

### Morphological observations and measurements of skin in testosterone-treated Hetian sheep

**Fig. 1:**
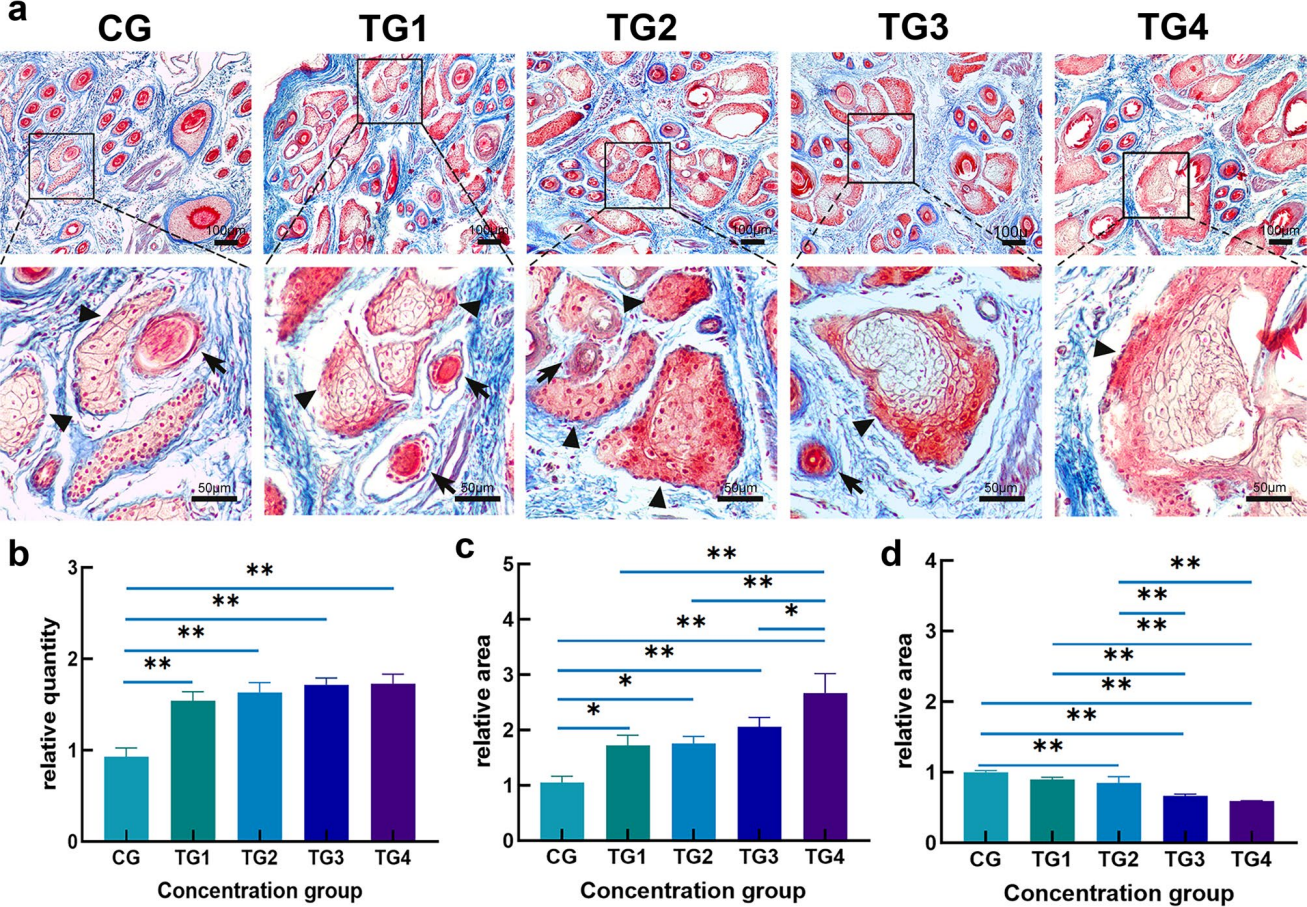
Masson staining analysis showing the impact of treatment with different concentrations of testosterone on the skin structure of Hetian sheep. (**a**) The skin structure of CG, TG1, TG2, TG3 and TG4 groups. The magnification of the upper image is 100 times, and the magnification of the lower image is 400 times; (**b**) Comparative analysis of SG counts between subject groups; (**c**) Comparative analysis of SG area between subject groups; (**d**) Statistical analysis of collagen fiber area in each group. Arrowheads indicate SGs, while arrows indicate hair follicles. Scale bars: the upper image: 100 μm; the lower image: 50 μm. *significant difference (*P* < 0.05), **Highly significant difference (*P* < 0.01).

The impact of testosterone treatment on the skin morphology of Hetian sheep was evaluated using Masson’s staining. Findings showed a significant increase in the number of SGs and their average area across all four testosterone-treated groups compared to the control group (*P* < 0.05, Fig. 1a). The control group had about 15 SGs per mm² in the microscopic field. In comparison, the treatment groups displayed an increase in SG count exceeding 1.45 times that of the control (Fig. 1b). Furthermore, the average SG area showed a trend of progressive enlargement corresponding with rising testosterone concentrations (Fig. 1c). However, the content of collagen fibers in the skin gradually decreased as testosterone levels increased (Fig. 1d).

### Screening of DEGs

**Fig. 2:**
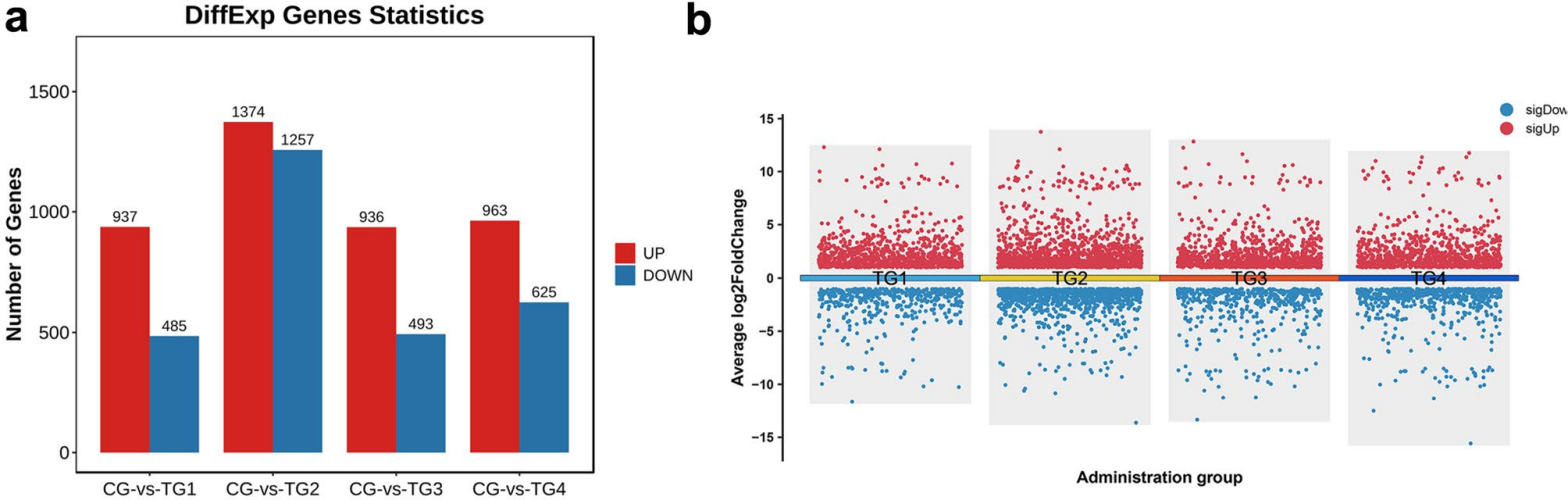
Comparative analysis of gene expression patterns in the skin of Hetian sheep between the TG groups and CG. (**a**) Number of DEGs between each TG and CG; (**b**) Volcano plot showing changes in DEGs for each TG compared with CG.

Gene expression differences in the skin of Hetian sheep were compared between the CG and the four testosterone treatment groups. The DEGs were identified using thresholds of |log2(FoldChange)| > 1 and a q-value < 0.05 (Fig. 2a-b), with results illustrated through volcano plots. The comparison of TG1 to CG revealed 937 upregulated and 485 downregulated genes, while TG2 versus CG showed 1,374 upregulated and 1,257 downregulated genes. In comparing TG3 and CG, 936 genes were upregulated and 493 downregulated, whereas TG4 versus CG had 963 upregulated and 625 downregulated genes. TG2 displayed the most pronounced alterations in gene expression profiles, indicating that a testosterone dose of 2 mg/kg body weight may represent an optimal level for investigating androgen effects on Hetian sheep skin.

### Common DEG trend analysis and GO enrichment analysis

**Fig. 3:**
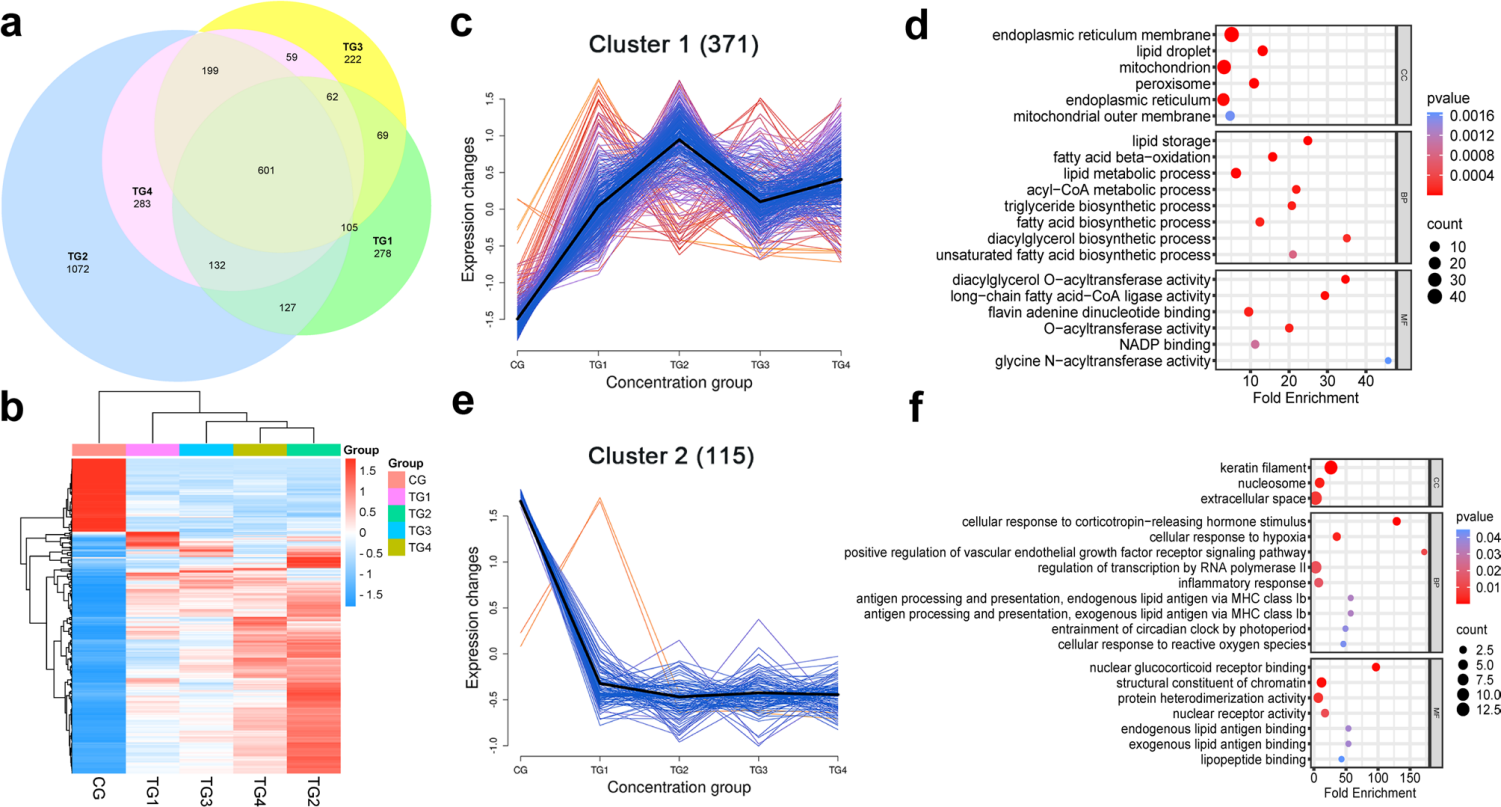
Analysis of DEGs, expression trends, and GO enrichment across the treatment groups. (**a**) Venn diagram of DEGs across the four treatment groups; (**b**) Heatmap showing the expression of the 486 identified genes; (**c**) Expression patterns of the 371 upregulated genes; (**d**) GO functional enrichment analysis of the 371 upregulated DEGs; (**e**) Expression patterns of the 115 down-regulated genes; (**f**) KEGG pathway enrichment analysis of the 115 down-regulated DEGs.

Venn diagrams were employed to evaluate the overlap of DEGs across the different sample groups (Fig. 3a). This analysis identified 601 common DEGs, including 486 annotated genes and 115 uncharacterized ones. The expression patterns of the 486 known genes were visualized through heatmaps (Fig. 3b). Subsequent expression trend analysis categorized these DEGs into upregulated and downregulated groups, revealing 371 genes with increased expression (Fig. 3c) and 115 genes with decreased expression (Fig. 3e). Moreover, GO analysis of the upregulated genes (Fig. 3d) showed significant enrichment in terms related to endoplasmic reticulum membrane (GO:0005789), lipid droplets (GO:0005811), mitochondria (GO:0005739), and fatty acid β-oxidation (GO:0006635) (*P* < 0.05), indicating that androgen treatment may impact lipid metabolism in the skin. For the downregulated genes (Fig. 3f), GO terms such as keratin filament (GO:0045095), cellular response to corticotropin-releasing hormone stimulus (GO:0071376), and nuclear glucocorticoid receptor binding (GO:0035259) were significantly enriched (*P* < 0.05).

### Effects of androgen on the expression of keratin intermediate filament genes

**Fig. 4:**
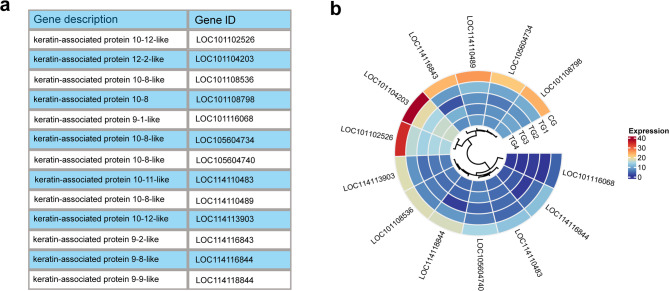
Results of keratin filament gene expression. (**a**) List of keratin filament genes; (**b**) Heatmap of keratin filament expression levels across the experimental groups.

The GO analysis of the downregulated genes showed significant enrichment in the keratin intermediate filament category (GO:0045095) (*P* = 9.3E-14), which includes 13 keratin-associated protein genes potentially involved in wool structure (Fig. 4a). Clustering analysis of gene expression across the groups (Fig. 4b) revealed that two genes, *LOC101102526* and *LOC101104203*, maintained the highest expression levels. However, partially suppressed, they remained active following androgen treatment. Furthermore, 9 genes showed clear inhibition of expression as androgen concentration increased. Among these, *LOC114118844* and *LOC114116843* initially exhibited decreased expression before showing a later increase, whereas the other seven genes demonstrated a steady decline in expression with rising androgen levels.

### Common DEGs in PPI network analysis

**Fig. 5:**
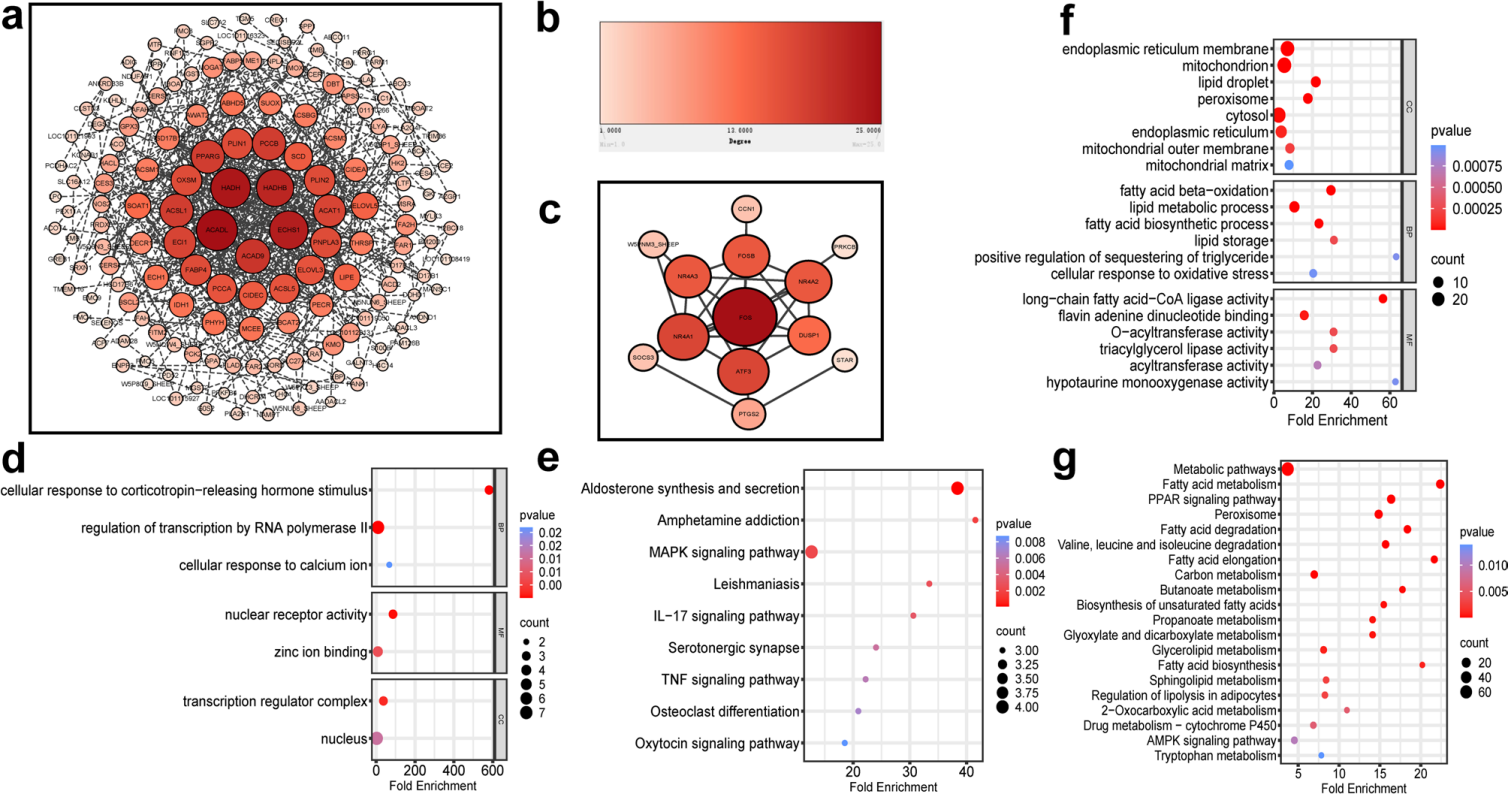
Construction and analysis of the DEG PPI network. (**a**) Protein interaction network of the upregulated DEGs; (**b**)The color intensity (light to dark) corresponds to Degree values (from 1 to 25), with larger values indicated by darker colors. Higher Degree values also suggest greater biological relevance; (**c**) Protein interaction network from the A network of the down-regulated DEGs; (**d**) GO analysis of down-regulated DEGs; (**e**) KEGG pathway analysis of down-regulated DEGs; (**f**) GO analysis of upregulated DEGs; (**g**) KEGG pathway analysis of upregulated DEGs.

Protein-protein interaction networks were analyzed separately for upregulated and downregulated genes, identifying interaction networks for 164 upregulated genes (Fig. 5a) and 13 downregulated genes (Fig. 5c), with their respective degree values illustrated in Fig. 5b. The functional roles of these gene sets within the PPI networks were explored through GO annotation and KEGG pathway enrichment analyses. GO analysis revealed that downregulated genes were significantly enriched in processes such as cellular response to corticotropin-releasing hormone stimulus (GO:0071376), regulation of transcription by RNA polymerase II (GO:0006357), among other biological processes (Fig. 5d) (*P* < 0.05). KEGG pathway analysis of the downregulated genes highlighted significant enrichment in the IL-17 signaling pathway (ko04657), MAPK signaling pathway (ko04010), aldosterone synthesis and secretion (ko04925), and other pathways (Fig. 5e) (*P* < 0.05).

**Fig. 6:**
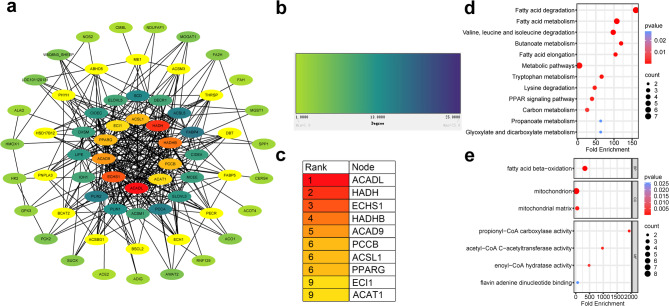
Hub gene analysis of upregulated DEGs. (**a**) Network of the top 10 hub genes and their associated genes; (**b**) Sub-network of surrounding genes, color-coded according to Degree values which ranged from 1 to 16; (**c**) Ranking of the 10 hub genes based on Degree values (18 to 25); (**d**) Functional enrichment analysis of the 10 hub genes was conducted using the GO database. (**e**) Pathway enrichment analysis of the 10 hub genes was performed using KEGG.

On the other hand, GO analysis of upregulated genes showed significant enrichment in cellular components and processes, including the endoplasmic reticulum membrane (GO:0005789), mitochondrion (GO:0005739), and lipid droplet (GO:0005811) (Fig. 5f) (*P* < 0.05). KEGG analysis of these upregulated genes revealed significant enrichment in pathways related to lipid metabolism, such as the PPAR signaling pathway (ko03320), fatty acid metabolism (ko01212), and general metabolic pathways (ko01100) (Fig. 5g) (*P* < 0.05). Moreover, degree-based ranking analysis of the upregulated DEGs identified the top 10 hub genes within the PPI network: *ACADL*, *HADH*, *ECHS1*, *HADHB*, *ACAD9*, *PCCB*, *ACSL1*, *PPARγ*, *ECI1*, and *ACAT1* (Figs. 6a–c). Subsequent GO and KEGG analyses of these hub genes demonstrated significant enrichment in biological processes such as fatty acid beta-oxidation (GO:0006635), mitochondrial function (GO:0005739), and acetyl-CoA C-acetyltransferase activity (GO:0003985) (Fig. 6d) (*P* < 0.05). KEGG pathway analysis further confirmed significant enrichment in fatty acid degradation (ko00071) and fatty acid metabolism (ko01212) pathways (Fig. 6e) (*P* < 0.05).

### GSEA analysis

**Fig. 7:**
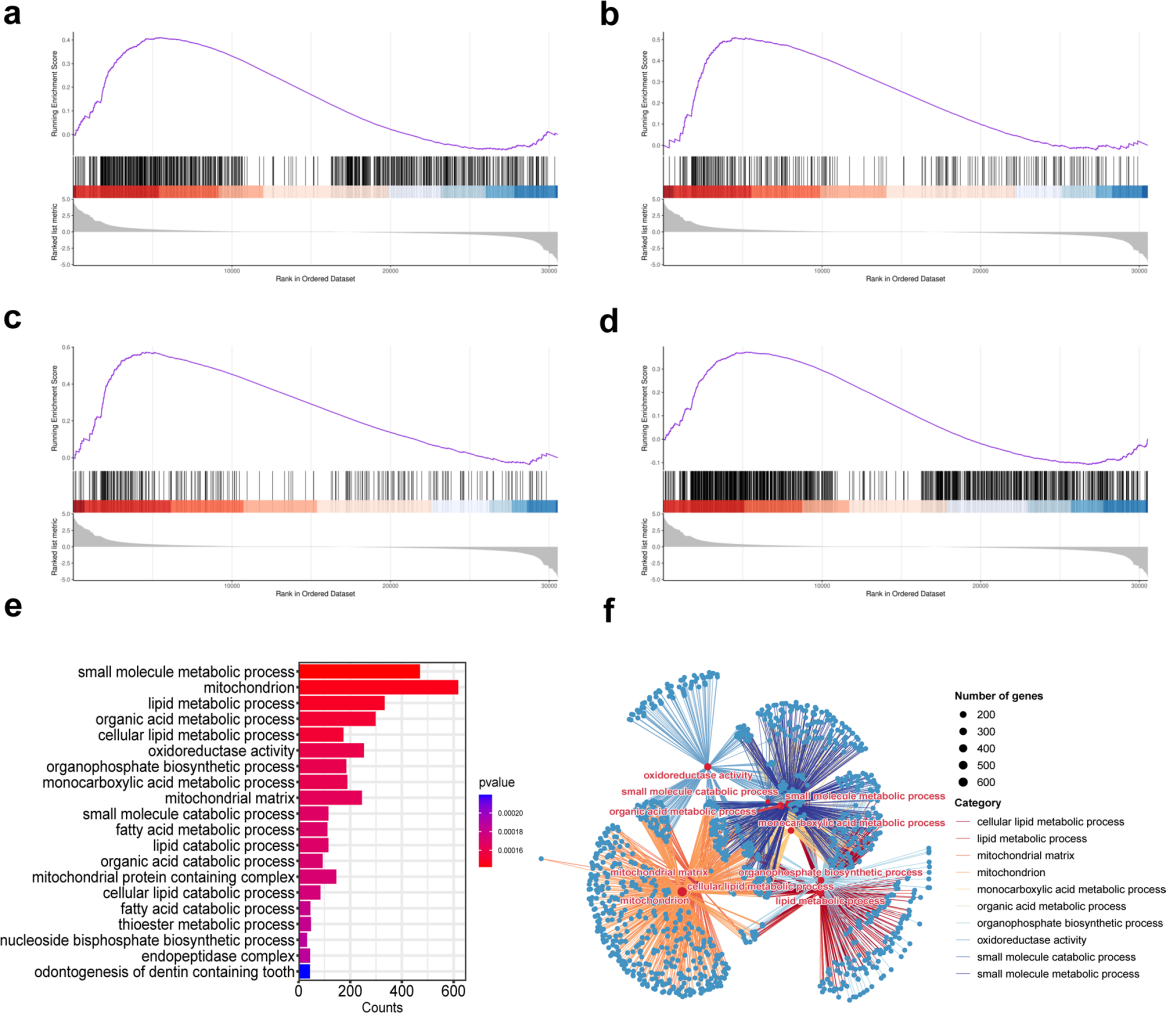
GO analysis and gene-pathway enrichment network analysis by GSEA for the TG2 group compared with the CG group. Significant up-regulation of (**a**) cellular lipid metabolic processes (*P* = 0.000152, NES = 1.421); (**b**) fatty acid metabolic processes (*P* = 0.000169, NES = 1.646); (**c**) lipid catabolic processes (*P* = 0.000169, NES = 1.833) and (**d**) lipid metabolic processes (*P* = 0.000148, NES = 1.306) in TG2; (**e**) Bar graphs were plotted using the 20 processes exhibiting the lowest FDR values; (**f**) Gene-pathway enrichment network analysis plots were constructed using the 10 processes exhibiting the lowest FDR values.

The GSEA of the entire gene set identified significant enrichment exclusively in the TG2 group compared to the CG group (|NES| > 1, *P* < 0.05, FDR < 0.25). In comparison, no meaningful differences were observed in the other treatment groups. Subsequent GO analysis via GSEA highlighted the top 20 biological processes with the lowest *P*-values, among which odontogenesis of dentin-containing tooth (GO:0042475) was significantly downregulated. In comparison, multiple lipid metabolism-related biological processes, including lipid metabolic process (GO:2001289), fatty acid metabolic process (GO:0006631), cellular lipid metabolic process (GO:0044255), and lipid catabolic process (GO:0016042) were significantly upregulated (Figs. 7a–e). Furthermore, gene-pathway enrichment network maps constructed from the 10 most significant biological processes revealed strong interconnections among these metabolic pathways (Fig. 7f).

**Fig. 8:**
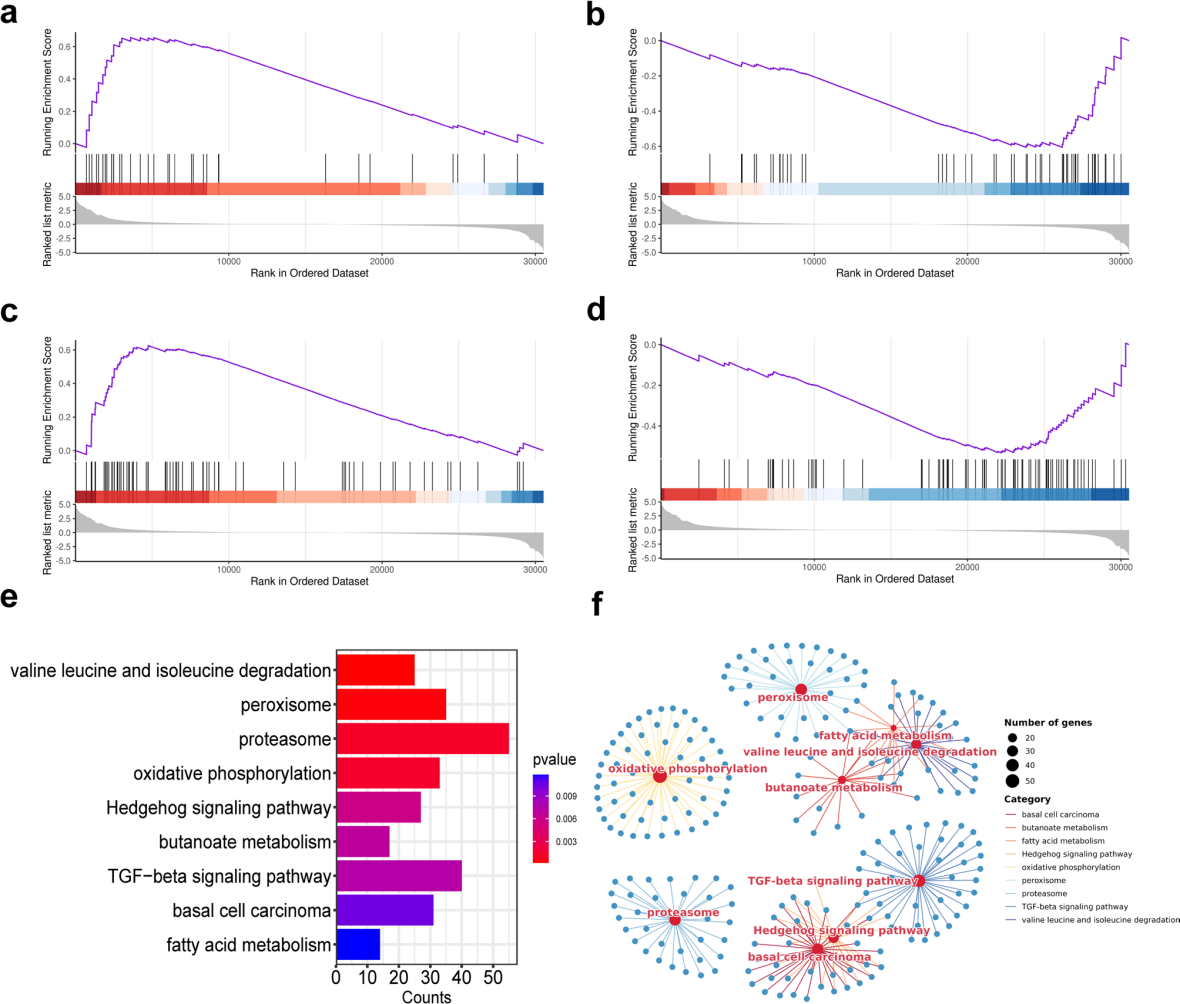
Analysis of KEGG pathways and gene-pathway enrichment networks by GSEA for the TG2 group compared with the CG group. (**a**) Fatty acid metabolism was significantly upregulated in TG2 (*P* = 0.011810, NES = 1.576); (**b**) Hedgehog signaling pathway was significantly decreased in TG2 (*P* = 0.005917, NES=−1.590); (**c**) Peroxisome pathway was enhanced considerably in TG2 (*P* = 0.000376, NES = 1.717); (**d**) TGF-beta signaling pathway was significantly decreased in TG2 (*P* = 0.007490, NES=−1.508); (**e**) Bar graphs showing all the apparent pathways; (**f**) Gene-pathway enrichment network analysis plot.

KEGG pathway analysis through GSEA corroborated these findings, showing upregulation of the fatty acid metabolic pathway (involving ACSL1, ALDH1B1, ACSL5, ACADL, ADH7, ECI1, ACAT1, HADH, ECHS1, HADHA, HADHB, EHHADH, ACAT2, and ACADVL), consistent with the GO results. On the other hand, the Hedgehog (GLI3, DHH, BMP7, SMO, GAS1, WNT5A, WNT6) and TGF-beta signaling pathways (CREBBP, SMAD3, THBS4, ACVR1, TFDP1, THBS3), both implicated in hair follicle growth and development, were suppressed. The peroxisome pathway (including ACSL1, PHYH, FAR2, FAR1, ACSL5), essential for lipid metabolism, was upregulated (Figs. 8a–e). Then, nine pathways with significant *P*-values were used to generate gene-pathway enrichment network maps (Fig. 8f).

### Verification by qRT-PCR

**Fig. 9:**
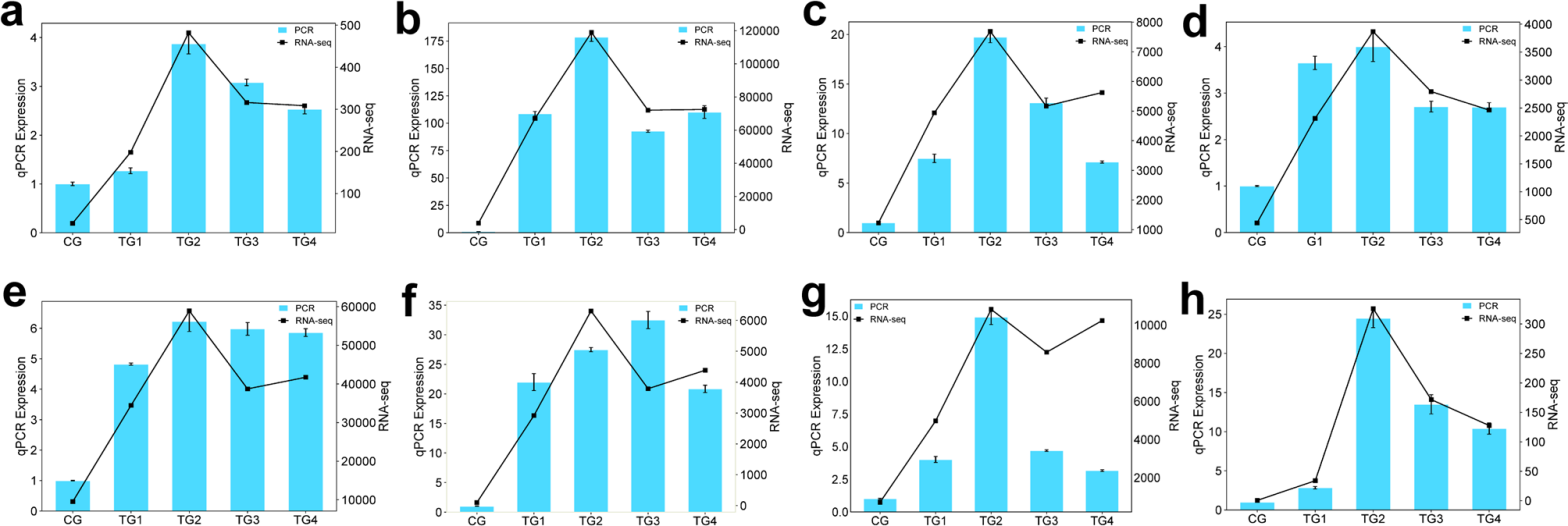
Verification of RNA-seq results by qRT-PCR. The left axis indicates the relative gene expression level (mean ± SEM), while the right axis shows RNA-seq Count values. Bar graphs and line charts show qPCR results and RNA-seq results, respectively.

The expression of eight prominently upregulated genes in the RNA-seq analysis was verified by qRT-PCR. Overall, the trends identified through qRT-PCR relative quantification aligned with the RNA-seq results (count values), confirming the reliability of the transcriptome sequencing data. (Fig. 9).

### Distribution of CERS4, andACSL1 expression in SGs

**Fig. 10:**
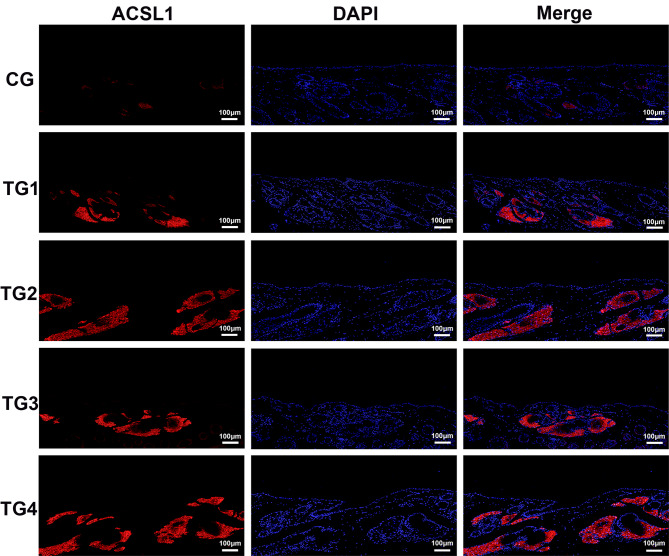
Immunofluorescence detection of ACSL1 in the SGs of treatment and control groups. Scale: 100 μm, magnification 100×.

**Fig. 11:**
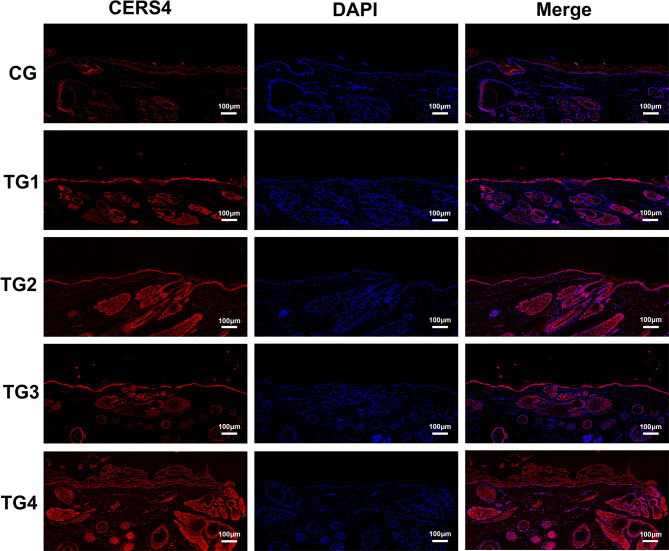
Immunofluorescence detection of CERS4 in the SGs of treatment and control groups. Scale: 100 μm, magnification: 100×.

**Fig. 12:**
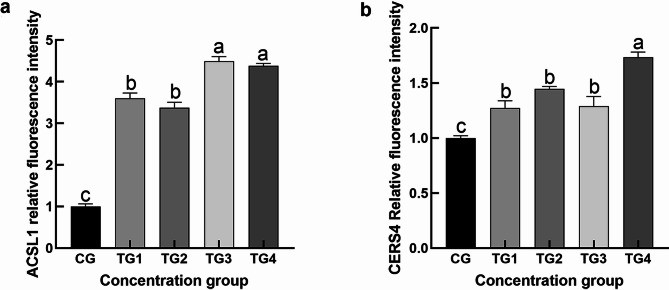
Fluorescence intensity analysis. (**a**) Relative fluorescence intensity of ACSL1 in the SGs of each group; (**b**) Relative fluorescence intensity of CERS4 in the SGs of each group. (mean ± SEM).

Immunofluorescence analysis demonstrated that ACSL1 exhibited the strongest positive signal in TG3 and TG4, while the weakest expression was observed in the CG. ACSL1 localization was restricted exclusively to SGs, with no detectable presence in the epidermis, hair follicles, or other skin appendages (Fig. 10). These results indicate that ACSL1 may serve as a specific marker for sebaceous gland cells in sheep. In comparison, CERS4 showed limited expression within the SGs of the CG group. However, all four testosterone-treated groups displayed significantly stronger positive staining (Fig. 11). Subsequent quantitative analysis of fluorescence intensity further confirmed that ACSL1 and CERS4 protein expression was significantly elevated across all treatment groups compared to CG (Figs. 12a–b).

## Discussion

Sebocytes in the skin can synthesize cholesterol, which is essential for cell membranes, the epidermal barrier, and sebum formation^11^. SG expansion and sebum secretion are androgen-dependent processes, with a slight increase in sebum production observed in males compared with females during puberty^12^. Individuals with androgen-induced acne exhibit increased production of T and DHT in their skin compared to unaffected individuals. Furthermore, elevated plasma levels of compounds like DHT biochemical indicators of androgen metabolism and activity in the skin have been observed in female patients with acne^13^. This study employed Hetian sheep as the experimental model to examine the effects of T on skin structure. Histomorphometric analyses were first conducted to evaluate alterations in skin morphology, followed by RNA-seq to explore the molecular mechanisms by which androgens modulate these morphological changes.

In this study, Hetian sheep were administered different concentrations of testosterone, and the primary findings revealed a significant increase in both the size and number of SGs in the skin correlating with rising testosterone levels. These results are consistent with previous studies demonstrating androgenic effects on mammalian skin. For instance, Iwata^14^ et al. treated castrated goats with subcutaneous T capsules and observed enlargement of their SGs, with the latter subsequently regressing to normal size after removing the T. Similarly, Ebling^15^ et al. reported that T treatment of castrated male rats prominently increased the secretion of sebaceous lipids. These findings are consistent with the results of the current study, indicating that androgens promote sebaceous gland enlargement and stimulate sebocyte proliferation and differentiation, therefore increasing sebum production.

Four groups of DEGs were identified by comparing testosterone-treated groups with the control, using defined threshold criteria. Venn diagram analysis revealed 601 DEGs commonly shared across all testosterone-treated groups relative to the control. The GO analysis of the up-and down-regulated genes (Fig. 3) highlighted distinct biological processes influenced by androgen treatment. Upregulated genes were significantly enriched in pathways related to fatty acid biosynthesis, suggesting that all T concentrations promoted lipid metabolism in the skin. On the other hand, downregulated genes were primarily associated with keratin filaments (GO:0045095) and specific cellular responses. Among these, 13 genes encoded keratin-associated proteins (KAPs) or structurally similar proteins. Since keratin and KAPs are key structural components of wool fiber^16^, the apparent down-regulation of KAPs in the presence of T suggests that androgens could influence the composition of wool structural proteins. Consistent with these observations, previous studies have shown that treatment with 10 nM DHT can downregulate hair follicle markers while upregulating genes involved in sebocyte differentiation in human SEBO662 AR + cells^9^. These findings suggest that androgens modulate hair characteristics and sebocyte differentiation by enhancing lipid metabolism. Transcriptomic analysis revealed that androgen exposure upregulates genes associated with sebaceous gland proliferation and differentiation while downregulating certain keratin-related genes involved in hair follicle structure. This raises the question of whether similar molecular and cellular mechanisms underlie the development of androgenic alopecia. Few studies to date have investigated the pathogenesis of androgenic alopecia by simultaneously considering the interplay between sebaceous glands and hair follicles.

The upregulated and downregulated DEGs were subjected to PPI network construction and GO and KEGG pathway enrichment analyses. Among the upregulated genes, ten key hub genes were identified: *ACADL*,* HADH*,* ECHS1*,* HADHB*,* ACAD9*,* PCCB*,* ACSL1*,* PPARγ*,* ECI1*, and *ACAT1*. These genes encode enzymes and regulatory proteins essential for adipocyte differentiation and lipid metabolism, underscoring their pivotal roles in these biological processes. GO enrichment analysis of the upregulated genes within the PPI network revealed a significant association with mitochondrial functions (GO:0005739). In particular, the hub gene *HADHB* encodes hydroxyacyl-CoA dehydrogenase, a vital subunit of the mitochondrial trifunctional protein complex, which is responsible for catalyzing the final three steps of long-chain fatty acid β-oxidation in mitochondria^17,18^. Additional upregulated genes enriched in mitochondrial lipid metabolism (GO:0005739) included *ECHS1*,* ACADL*, and *ACSM1*, all participating in mitochondrial fatty acid synthesis and degradation. ECHS1 (Enoyl-CoA Hydratase, Short Chain 1) is a mitochondrial matrix enzyme that plays a crucial role in the catabolism of various amino acids and fatty acids, highlighting its importance in cellular energy metabolism^19–22^.

In comparison, *ACADL* catalyzes the initial step in long-chain fatty acid oxidation and is vital for regulating lipid metabolism^23,24^. Studies in goats have demonstrated that *ACADL* is expressed at significantly higher levels in sebaceous glands than in other tissues. Moreover, it facilitates subcutaneous adipocyte differentiation by upregulating key regulatory genes, including *PPARγ*^25^. Finally, metabolic dysfunction of Acyl-CoA Synthetase Medium-Chain Family Member 1 (ACSM1) has been implicated in inducing mitochondrial oxidative stress and promoting lipid peroxidation^26^. These findings suggest that androgens significantly impact mitochondrial lipid metabolism by regulating the expression of mitochondrial catalytic enzymes, increasing the skin’s lipid storage capacity. It is further proposed that androgens may support energy production required for sebaceous gland cell proliferation and subcutaneous adipocyte differentiation by stimulating mitochondrial lipid metabolism. This mechanism likely contributes to the increased size and number of sebaceous glands following androgen treatment, enabling the skin to accumulate and secrete more lipids and consequently modifying its ecological landscape. Based on these findings, targeting mitochondrial lipid metabolism regulated by androgens presents a promising avenue for dermatology and cosmetic science advancements. For instance, developing localized antagonists could selectively inhibit lipid peroxidation within sebaceous gland mitochondria, reducing excessive sebum production without the systemic side effects associated with traditional anti-androgen therapies. However, drugs designed to activate mitochondrial lipid synthesis pathways could benefit conditions such as atopic dermatitis or age-related lipid deficiency by increasing epidermal lipid production and restoring local sebaceous gland metabolic balance.

PPI network analysis of the downregulated genes highlighted *FOS* and *ATF3*, which are strongly linked to lipid metabolism, wound healing, and immune responses. *FOS*, in particular, influences wound healing by regulating *MT3* expression, and research on salamander skin has shown that inhibiting *FOS* delays the wound-healing process^27^. Further evidence indicates that androgens, recognized as negative regulators of wound healing, are associated with delayed acute wound healing in older males who have elevated testosterone levels^28^. Collagen fibers are essential for tissue integrity, wound healing, and tensile strength, and they support homeostasis and epithelialization^29^. During inflammatory responses, type I and type IV collagen fragments serve as chemotactic signals for neutrophils, promoting immune activity and phagocytosis while influencing gene expression^30^. In this study, Masson staining revealed that androgens could prominently reduce skin collagen fiber density, suggesting they hinder wound healing by suppressing collagen production. Further, transcription factors, such as FOS and ATF3, can regulate adipocyte behavior and differentiation^31^, with ATF3 specifically inhibiting adipocyte differentiation by down-regulating the expression of C/EBPα^32^ or PPARγ^33^. These findings indicate that androgens influence lipid metabolism and adipocyte function while hindering wound healing by suppressing collagen synthesis and altering inflammatory responses, ultimately leading to delayed tissue repair. This insight may be particularly relevant for male trauma patients with elevated androgen levels. Monitoring serum testosterone levels in these patients could allow for timely and tailored adjustments in the use of androgen inhibitors to prevent delayed postoperative recovery.

PPI network analysis of the upregulated genes revealed 10 hub genes, six of which were linked to mitochondria (GO:0005739) according to GO analysis. These hub genes encode catalytic enzymes and regulatory proteins essential for lipid metabolism and adipocyte differentiation while also playing significant roles in lipid storage. GO and KEGG analyses indicated that these genes are primarily involved in mitochondrial functions and fatty acid metabolic pathways, underscoring the close relationship between mitochondrial activity and lipid metabolism and the pronounced impact of androgens on both processes. Among the hub genes, Acyl-CoA Synthetase Long-Chain Family Member 1 (ACSL1) belongs to the Acyl-CoA synthase family, essential for lipid metabolism^34^. ACSL1 also functions as a key regulator in adipocytes. Studies on bovine adipocytes have shown that overexpression of ACSL1 results in elevated levels of saturated fatty acids and polyunsaturated fatty acids (PUFAs), accompanied by increased lipid droplet accumulation^35^. The androgen-induced upregulation of this gene can even enhance the lipid storage capacity of SGs and subcutaneous adipocytes.

Furthermore, androgens can stimulate the expression of PPARγ. For instance, in the skin, it was found to regulate a network of genes associated with cell proliferation, differentiation, and inflammatory responses^36,37^. Other studies reported that PPARγ regulated adipocyte differentiation and lipid metabolism in adipocytes^38^, with its up-regulation shown to enhance the de novo synthesis of lipids, such as fatty acids and phospholipids, in mass spectrometry-based lipidomics studies^39^. Thus, the upregulation of catalase genes involved in fatty acid metabolism may be driven by increased PPARγ expression. Furthermore, studies in mice have demonstrated that PPARγ deficiency results in complete fat atrophy and the absence of sebaceous glands^40^. Therefore, androgens may stimulate sebaceous gland proliferation, sebocyte differentiation, and sebum secretion in Hetian sheep by upregulating PPARγ. Current evidence also supports that androgens enhance sebocyte differentiation and lipid accumulation via PPARγ-mediated pathways. Both androgens and other PPARγ ligands can potentially increase sebum production, which may play a role in developing seborrheic dermatitis^41,42^. Acne patients have lipid peroxides in their sebum due to the peroxidation of SGs’ specific lipids by their keratinocytes^43^. These peroxides activate PPARα and PPARγ, triggering ligand-receptor complexes that regulate sebocyte proliferation, differentiation, lipid synthesis, and related pathways^44–46^. As a result, upregulation of PPARγ may affect the transcription of other genes involved in lipid metabolism, promoting the proliferation and differentiation of sebaceous gland cells and adipocytes, boosting their lipid storage capacity, and disrupting skin homeostasis.

KEGG analysis from GSEA indicated a clear downregulation of the Hedgehog and TGF-beta signaling pathways. Research in mice demonstrated that Hedgehog signaling in fibroblasts promotes hair growth and cell proliferation, while the TGF-beta pathway, acting downstream of Hedgehog signaling in the dermis, mediates excessive hair growth. This role was further supported by studies showing that pharmacological inhibition of TGF-beta reversed the hyperactivation phenotype^47^. These findings suggest that downregulating the Hedgehog and TGF-beta pathways may inhibit hair growth, implying that androgens could suppress hair growth by targeting these pathways and decreasing keratin expression. Subsequent GO analysis of GSEA revealed significant enrichment in metabolic processes, especially lipid metabolism, which were consistently upregulated. These results align with the GO analysis of upregulated genes and their PPI networks. Furthermore, GO analysis of GSEA showed that seven of the top ten biological processes with the lowest P-values shared a substantial overlap of genes, indicating extensive inter-regulation among metabolism-related pathways influenced by androgens. This observation further supports the identification of lipid metabolism genes as the most prominently upregulated hub genes.

The qRT-PCR validation of eight selected genes confirmed their increased expression following androgen treatment, which was consistent with the RNA-seq results. Furthermore, immunofluorescence staining of two genes showed a clear increase in fluorescence intensity after androgen exposure, supporting the transcriptomic data. *ACSL1* acts as a regulator in adipocytes; studies in bovine adipocytes have shown that overexpression of *ACSL1* results in elevated levels of saturated fatty acids^35^. Another gene, *CERS4*, is crucial for maintaining the homeostasis of sebaceous gland units, with its knockdown altering sebum lipid composition and leading to more viscous sebum secretion^48^. Deleting the *CERS4* gene can disrupt skin lipid metabolism and impair epidermal barrier function, ultimately contributing to eczematous skin barrier disorders^49^. Thus, these two key genes likely regulate sebum secretion and the proliferation and differentiation of sebaceous glands in sheep. Androgens may alter the lipid composition of Hetian sheep sebum by upregulating *ACSL1* and *CERS4* expression, influencing sebaceous gland lipid metabolism, modifying the skin’s lipid profile, and impacting the barrier function of sheep skin.

In recent years, numerous studies have integrated transcriptome analysis with artificial neural networks (ANNs), primarily to build models linking gene expression profiles with biological traits^50,51^ and to screen disease samples. ANNs demonstrate improved performance in handling complex data modeling and prediction tasks^52^. After identifying hub genes through transcriptome analysis, researchers have applied five different methods, including ANN, to differentiate disease samples from normal ones, with ANN achieving the highest accuracy^53^. Further, we investigate the mechanisms by which androgens affect the skin. We plan to use ANN to develop a model that correlates androgen-induced changes in gene expression with the extent of disruption to skin homeostasis, aiming to enhance both the predictive accuracy and biological interpretability of the model.

## Conclusion

This study investigated the effects of varying androgen concentrations on Hetian sheep skin using morphological and transcriptomic approaches. Morphological findings demonstrated that androgen treatment significantly increased the size and number of sebaceous glands compared to the control group. Transcriptomic analyses revealed that androgens significantly stimulated lipid metabolism in the skin, particularly emphasizing mitochondrial lipid metabolism. The results provide preliminary evidence that androgens enhance mitochondrial lipid processing, supply energy for cellular proliferation, and boost the lipid storage capacity of sebaceous glands. This research advances the theoretical understanding of how androgen-driven alterations in skin lipid metabolism may contribute to developing novel therapeutic strategies for various skin disorders. By integrating multi-scale data, the study deepens insights into the complex interplay between androgens and skin biology and lays the groundwork for more precise, targeted treatments. Furthermore, identifying specific genetic and molecular pathways influenced by androgens in sheep skin offers a valuable model for investigating analogous human conditions, potentially facilitating the creation of more effective and safer therapies.

## Electronic supplementary material

Below is the link to the electronic supplementary material.

Supplementary Material 1

Supplementary Material 2

Supplementary Material 3

## Data Availability

The resulting original cleaned data were deposited in the NCBI SRA database (https://www.ncbi.nlm.nih.gov/) under accession number PRJNA1202934.
